# Highly Retentive, Anti‐Interference, and Covert Individual Marking Taggant with Exceptional Skin Penetration

**DOI:** 10.1002/advs.202201497

**Published:** 2022-06-24

**Authors:** Lianggen Zhong, Jiguang Li, Baiyi Zu, Xiaodan Zhu, Da Lei, Guangfa Wang, Xiaoyun Hu, Tianshi Zhang, Xincun Dou

**Affiliations:** ^1^ Xinjiang Key laboratory of Explosives Safety Science Xinjiang Technical Institute of Physics & Chemistry Chinese Academy of Sciences Urumqi 830011 China; ^2^ Center of Materials Science and Optoelectronics Engineering University of Chinese Academy of Sciences Beijing 100049 China

**Keywords:** fluorescent, optical sensing, skin marking, taggants, upconversion nanoparticles (UCNPs)

## Abstract

The development of high‐performance individual marking taggants is of great significance. However, the interaction between taggant and skin is not fully understood, and a standard for marking taggants has yet to be realized. To achieve a highly retentive, anti‐interference, and covert individual marking fluorescent taggant, Mn^2+^‐doped NaYF_4_:Yb/Er upconversion nanoparticles (UCNPs), are surface‐functionalized with polyethyleneimine (PEI) to remarkably enhance the interaction between the amino groups and skin, and thus to facilitate the surface adhesion and chemical penetration of the taggant. Electrostatic interaction between PEI_600_‐UCNPs and skin as well as remarkable penetration inside the epidermis is responsible for excellent taggant retention capability, even while faced with robust washing, vigorous wiping, and rubbing for more than 100 cycles. Good anti‐interference capability and reliable marking performance in real cases are ensured by an intrinsic upconversion characteristic with a distinct red luminescent emission under 980 nm excitation. The present methodology is expected to shed light on the design of high‐performance individual marking taggants from the perspective of the underlying interaction between taggant and skin, and to help advance the use of fluorescent taggants for practical application, such as special character tracking.

## Graphical abstract

Here, it is demonstrated that a highly retentive, anti‐interference, and covert individual marking upconversion nanoparticles‐based taggant can be achieved by enhancing the electrostatic interaction between polyethyleneimine and lipid‐induced effective skin‐penetrating. It brings about high marking efficiencies on skin, hair, and cloth, as well as superior anti‐interference capability in complex scenarios with simultaneously existing fluorescent and colorful substances.

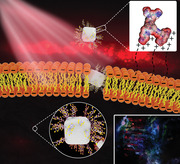

## 1 Introduction

Taggants, which have rapidly emerged as preferable materials for physically or chemically marking an item for unique identification, are used extensively in the fields of anticounterfeiting,^[^ ^1^ ^]^ cell tracking,^[^ ^2^ ^]^ property marking,^[^ ^3^ ^]^ suspect monitoring,^[^ ^4^ ^]^ explosives or illegal narcotics tracking and identification,^[^ ^5^ ^]^ etc. To date, a variety of taggants have been explored using various principles, such as fluorescent taggants,^[^ ^6^ ^]^ photochromic and thermochromic inks,^[^ ^7^ ^]^ magnetic inks,^[^ ^8^ ^]^ radioactive isotopes,^[^ ^9^ ^]^ and Raman‐active components.^[^ ^10^ ^]^ Among them, fluorescent taggants are widely developed due to the merits of invisibility under natural lighting conditions and intense fluorescent emission upon an appropriate excitation. Moreover, compared to traditional tagging techniques, fluorescent taggants are inexpensive and have the potential to be widely applicable. Currently, fluorescent taggants are dominantly explored through markings in the visible‐wavelength region, including mainly organic fluorescent dyes,^[^ ^11^ ^]^ semiconductor quantum dots (QDs),^[^ ^12^ ^]^ 2D metal‐organic framework.^[^ ^13^ ^]^ Most of the reported fluorescent taggants employ the downconversion fluorescence mechanism, which emit low energy fluorescence when excited by high energy light. Fluorescent taggants are hampered by interference when other components, such as dust, skin tissue, fluorescent dyes, or plants,^[^ ^14^ ^]^ fluoresce upon UV excitation. This interference renders fluorescent taggants unreliable, especially in complex application sceneries. Many challenges must be addressed in order for fluorescent taggants to employ for practical applications. In particular, new mechanisms to explore emerging fluorescent taggants should be emphasized.

Near‐infrared (NIR) light is a promising excitation source for a variety of applications because of its minimal scattering, lower absorption, deep penetration, and low phototoxicity.^[^ ^15^ ^]^ Rare‐earth upconversion nanoparticles (UCNPs), with their unique advantage of converting lower energy NIR light into visible emissions based on a process termed upconversion, are among the most representative fluorescent materials. It has been reported that UCNPs exhibit many distinguished properties, including high signal‐to‐noise ratio,^[^ ^16^ ^]^ low autofluorescence,^[^ ^17^ ^]^ excellent photochemical stability,^[^ ^18^ ^]^ broadly tunable emission colors,^[^ ^19^ ^]^ low toxicity,^[^ ^20^ ^]^ and deep light penetration in biological specimens,^[^ ^21^ ^]^ and thus are highly attractive for a range of applications, such as bioimaging,^[^ ^20^, ^22^ ^]^ molecule tracking,^[^ ^23^ ^]^ ultrasensitive bioassays,^[^ ^24^ ^]^ labeling,^[^ ^25^ ^]^ barcoding,^[^ ^26^ ^]^ and anticounterfeiting.^[^ ^27^ ^]^ Whether UCNPs could emerge as a new generation of nontoxic, broadly applicable skin taggant that is not susceptible interference from various fluorescent substances is still unknown. Furthermore, it should be noted that taggants are used to provide strong physical evidence to uniquely identify individuals, whether directly through individual marking or indirectly through taggant transfer. Although this association could also be realized by developing encoding materials,^[^ ^28^ ^]^ including botanically derived DNA oligonucleotides^[^ ^29^ ^]^ on cash or valuables, peptides^[^ ^30^ ^]^ on skin, or rare elements combined in unique formulations^[^ ^31^ ^]^ embedded in items, the decoding process is inevitably tedious. One alternative to strengthen this association could be to extend the marking time with better retention capability using an anti‐exfoliating design to avoid interference stemming from daily activities and metabolization. The penetration of taggant into the outermost layer of skin, the stratum corneum, would protect the marking imprint from external interference^[^ ^32^ ^]^ and still be trackable despite epidermal metabolization and skin growth.^[^ ^33^ ^]^ Therefore, permeation of the taggant into the stratum corneum is of vital importance, and can be fulfilled via the effective surface functionalization of UCNPs.^[^ ^23b,23c^ ^]^ Improving marking performance is crucial in order to fully utilize the emitted optical signal from the penetrated UCNPs. A facile but efficient methodology to build a unique association by employing UCNPs to superficially penetrate skin with high‐retention capability presents a significant but worthwhile challenge, because of the great demand for special character tracking.

Here, a highly retentive, anti‐interference, and covert individual marking UCNP taggant was designed by the surface functionalization of Mn^2+^‐doped NaYF_4_:Yb/Er UCNPs with polyethyleneimine (PEI) to form PEI‐UCNPs. Because of the electrostatic interaction between the positively charged amino group in PEI and the negatively charged lipid in skin, a remarkable penetrating efficacy of PEI‐UCNPs deep inside the epidermis to a depth up to 144.6 µm was achieved. Excellent marking patterns with distinct red luminescent emission could be observed on skin with the naked‐eye under 980 nm excitation even after robust washing for 100 cycles and assisted with soap, organic solvent, as well as vigorous rubbing. Exceptional anti‐interference capability for PEI‐UCNPs was demonstrated by negligible interference on its marking performance in the presence of >14 types of fluorescent substances and daily cosmetics. Furthermore, excellent marking performance in a real case was demonstrated by good retention of labeled fingerprints, completely undisturbed discrimination of the target pattern hidden in a colorful tattoo, as well as thorough hair and cloth penetration.

## 2 Results and Discussion

**Figure 1:**
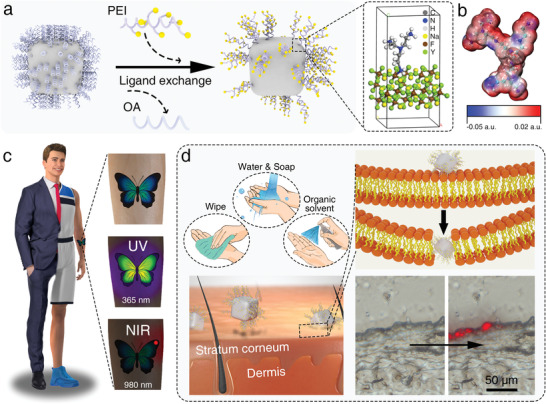
Schematic illustrations of the design of the PEI‐UCNPs for skin marking. a) The preparation process of the PEI‐UCNPs, NaYF_4_ cubic structure with PEI as ligand. b) The electrostatic potential distribution in PEI. The c) anti‐interference capability introduced by the upconversion strategy and d) highly retentive capability brought by the effective penetration of PEI‐UCNPs in skin due to the electrostatic interaction between the positively charged amino group in PEI and the negatively charged lipid in the skin.

The taggant, PEI‐UCNP, was obtained through a modified solvothermal method with Mn^2+^‐doped NaYF_4_:Yb/Er using PEI in place of the oleic acid ligand (**Figure** **1**a). It should be noted that PEI could be firmly bound to UCNP via coordination between the ‐NH_2_ group and the Y(III) ions. Furthermore, this design rendered a positive charge to the PEI amino group, which was verified by electrostatic potential distribution analysis (Figure 1b). It is expected that PEI‐UCNPs can effectively avoid fluorescence interference from contaminants, which inevitably appears under UV light and is covert due to the upconversion design (Figure 1c). Specifically, a butterfly tattoo pattern made with pigments mixed with fluorescent dyes and the upconversion taggant produced colorful fluorescence after excitation at 365 nm with no interference in the upconversion taggant marked area, which was excited only at 980 nm and presented a red emission.

A high‐performance taggant should be washproof and capable of highly retentive marking on individuals; thus PEI, which possesses multiple positively charged amino groups, was chosen as the functional ligand. The ‐NH_2_ group of PEI can interact with ‐COOH, ‐CO‐NH_2_, ‐OH, and imidazole groups on the skin surface to enhance its skin retention through chemical reactions and physical interactions, such as hydrogen bonding and electrostatic attractions. In addition, PEI is a safe and efficient permeation enhancing additive that can perturb lipid bilayers via fluidization, polarity alteration, phase separation, or lipid extraction,^[^ ^34^ ^]^ and thus help PEI‐UCNPs to penetrate the stratum corneum layer (Figure 1d). The synergistic effect of the interaction with skin and chemical penetration endows PEI‐UCNPs with promising marking efficiency, even after robust scrubbing and vigorous epidermis metabolism.

**Figure 2:**
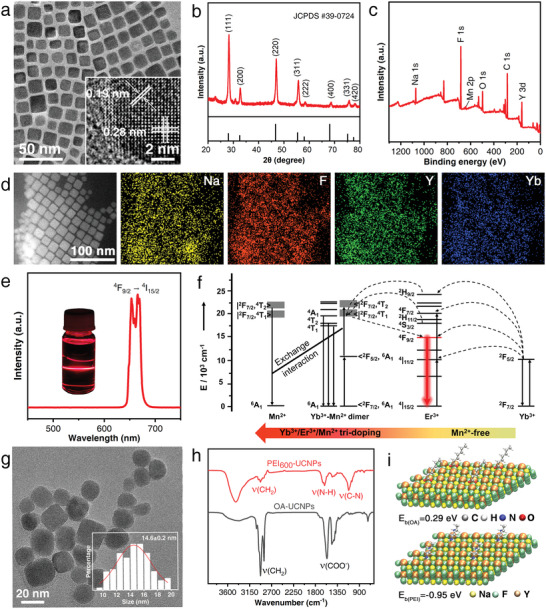
Characterization of the optimized Mn^2+^‐doped NaYF_4_:Yb/Er nanoparticles: a) TEM and HRTEM image (inset). b) XRD pattern (red curve) in comparison with the standard peaks of cubic phase NaYF_4_ (vertical black lines, JCPDS #39‐0724). c) XPS spectrum. d) Elemental mappings of Na, F, Y, and Yb ions by TEM. e) Upconversion emission spectra and the corresponding luminescent photograph (inset). f) Schematic energy level diagram showing the corresponding upconversion mechanism. g) TEM image and size distribution (inset) of the PEI_600_‐UCNPs. Data are shown as mean ± s.d. (*n* = 100). h) FT‐IR spectra of PEI_600_‐UCNPs (red) and OA‐UCNPs (black). i) Configuration of OA (upper) and PEI (bottom) bonding on the (111) surface of NaYF_4_.

Codoping the NaYF_4_:Yb/Er UCNPs with Mn^2+^ ions is important to the selective enhancement of red upconversion emission. A molar ratio of 50% Mn^2+^ in the precursor compared to the total Y amount, including Yb and Er, processes the most intense red emission. The average nanoparticle sizes in the whole doping range lie in 13.8–22.3 nm (Figure S1, Supporting Information), which has a neglectable influence on the emission but facilitates the penetration of particles into the skin. Transmission electron microscopy (TEM) shows that the oleic acid‐coated Mn^2+^‐doped NaYF_4_:Yb/Er UCNPs (OA‐UCNPs) with optimized Mn^2+^ concentration have a highly monodispersed cubic morphology (**Figure** **2**a) with an average diameter of 12.6 ± 0.1 nm after statistically evaluating 100 UCNPs (Figure S2, Supporting Information). High‐resolution TEM (HRTEM) image indicates that OA‐UCNPs exhibit good crystallinity, and the lattice spaces of 0.28 and 0.19 nm corresponding to the (200) and (220) directions of cubic phase NaYF_4_, respectively, confirmed the successful fabrication of cubic phase, high‐quality OA‐UCNPs. The main X‐ray diffraction (XRD) peaks are sharp and can be assigned to the standard cubic phase of NaYF_4_ (JCPDS #39‐0724), indicating that Mn^2+^ doping has no significant influence on crystallinity or cubic phase compared with the undoped UCNPs (Figure 2b and Figure S1i, Supporting Information). Corresponding X‐ray photoelectron spectroscopy (XPS) verified the presence of Na, Y, F, and Mn (Figure 2c). Compared with the spectrum of UCNPs without Mn^2+^ doping, the two peaks located at 642.14 and 654.02 eV can be attributed to the binding energy of Mn 2p_3/2_ and Mn 2p_1/2_,^[^ ^35^ ^]^ respectively (Figure S3, Supporting Information), indicating the successful doping of Mn at the site of Y. In addition, from large‐scale elemental mapping analysis on TEM, the distributions of Na, F, Y, and Yb are uniform in the entire observed area (Figure 2d), indicating the controllable synthesis of the doped UCNPs. Er and Mn could not be observed due to their limited incorporation into the UCNP lattice although element Mn is introduced in a relatively very high ratio in the precursor.

The upconversion luminescence spectrum collected under irradiation at 980 nm exhibits a distinct visible band at 635–690 nm stemming from ^4^F_9/2_→^4^I_15/2_ transitions of Er^3+^ ions, and this red emission can be clearly observed with the naked eye (Figure 2e). It should be noted that this red emission is selectively enhanced compared to the relatively weak green emission. It is considered that this prominent selective enhancement is caused by the codoped Mn^2+^ ions, which may originate from the sensitization via the Yb^3+^–Mn^2+^ dimer complex (Figure 2f). Thus, it is confirmed that the introduction of Mn^2+^ ions is essential for the bright red emission, which is much more conspicuous and reliably discernable from other background colors.

After exchanging the oleic acid ligand with PEI, typically with a molecular weight of 600, the PEI_600_‐UCNPs were further characterized by TEM, which revealed essentially unaffected nanoparticle morphology; statistical analysis of the TEM image indicated the average diameter increased to ≈14.6 ± 0.2 nm due to PEI modification (Figure 2g). Fourier transform infrared (FT‐IR) spectra confirmed this grafting with the appearance of absorption peaks at 1610 and 1121 cm^–1^, corresponding to the bending vibration of N‐H and stretching vibration of C‐N, respectively (Figure 2h and Figure S4, Supporting Information).^[^ ^36^ ^]^ Furthermore, coordination of PEI on UCNPs is more stable thermodynamically than that of OA,^[^ ^37^ ^]^ with a preferentially negative bonding energy of −0.95 eV for PEI (Figure 2i and Table S1, Supporting Information), demonstrating firm binding of PEI on UCNPs which is favorable for PEI‐induced dragging.

**Figure 3:**
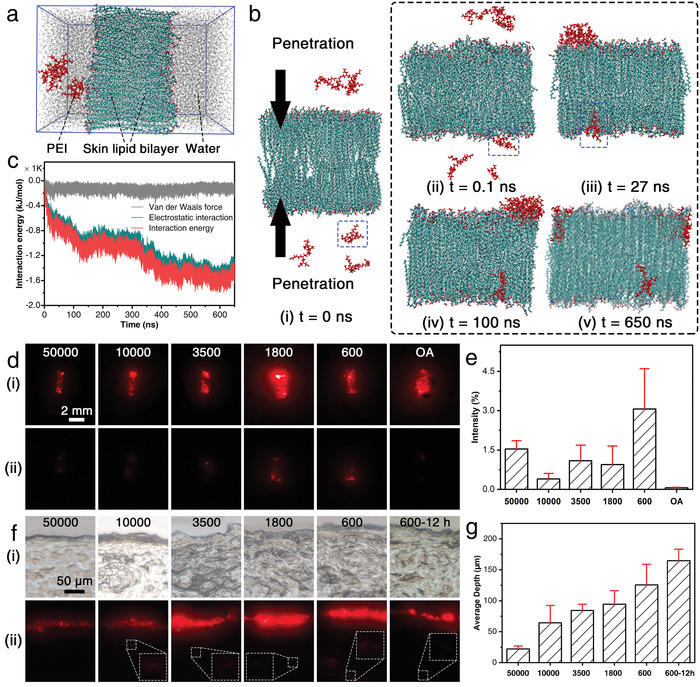
a) Density profiles of PEI and water along the normal direction of the skin lipid bilayer. b) Simulation snapshots of the penetration process over time. c) Interaction energy with time. d) Comparison of luminescent images of PEI‐UCNPs with different molecular weights of PEI and OA‐UCNPs markings on porcine skin i) before and ii) after washing under 980 nm excitation. e) The corresponding luminescence retention ratios after washing. Data are shown as mean ± s.d. (*n* = 3). f) Cross‐sectional images of porcine skin i) under natural light illumination and ii) under 980 nm excitation. g) Average penetration depth of various PEI‐UCNPs. Data are shown as mean ± s.d. (*n* = 5).

To validate the proposed strategy that surface modulation of UCNPs with PEI can form electrostatic interactions that enhance the skin retention of the luminescent tag and benefit from its chemical penetration, molecular dynamics (MD) simulations were carried out to elucidate the permeation mechanism and the interaction of PEI‐UCNPs through the stratum corneum layer. A simulation model was constructed via the three most abundant skin lipids, namely, free fatty acids, ceramides, and cholesterol, with an equimolar ratio to mimic a real stratum corneum layer (**Figure** **3**a and Figure S5, Supporting Information). To clearly visualize and understand the translocation of PEI during the penetration process, simulation snapshots (water molecules not shown for clarity) were monitored over time (Figure 3b). At *t* = 0, PEI is situated at the top without any contact with the skin bilayer surface (Figure 3b‐i). As time lapses, PEI quickly reaches and interacts with the bilayer surface (*t* = 0.1 ns, Figure 3b‐ii), due to favorable interactions between the polar moieties of PEI and the hydrophilic bilayer surface. During the interval of 0.1 and 27 ns (Figure 3b‐iii), many PEI moieties are adsorbed on the lipid layer. Additional PEI molecules penetrate the skin bilayer at 100 ns (Figure 3b‐iv), with all PEI moieties inside the bilayer at 650 ns (Figure 3b‐v), confirming that surface modulation of UCNPs with PEI is conducive to chemical penetration.

To quantitatively explain why PEI can penetrate, the interaction energy between PEI and skin lipids was evaluated (Figure 3c). Electrostatic interactions play a dominant role in interaction energy, whereas the van der Waals force remains basically unchanged over time. Initially (*t* = 0 ns), there is no interaction between PEI and the skin surface as PEI is distanced from the skin surface. Then, the interaction dramatically increases in the first 125 ns, indicating the fast penetration of PEI into the bilayer. However, the penetration slows down and plateaus from 125 to 330 ns, possibly due to the aggregation of PEI on the surface of the bilayer, which blocks the penetration channel. Next, the interaction energy decreases gradually, and the penetration reaches equilibrium from 300 to 650 ns, indicating that all the PEI has penetrated the skin lipids. Thus, the dominant negative energy resulting from the strong electrostatic interaction between PEI and the lipid greatly favors the chemical penetration of PEI into the skin.

To assess the influence of molecular weight of PEI on the marking performance, a set of PEIs with molecular weights ranging from 600 to 50 000 was grafted to UCNPs and was marked on porcine skin. Red luminescence could be clearly observed in a 1 mm × 3 mm rectangular area for all the UCNP‐marked skin tissues with or without PEI grafting (Figure 3d‐i). After washing with wet cotton swabs using liquid soap for 30 s, the red emissions for PEI_600_‐UCNPs and PEI_1800_‐UCNPs were most prominent (Figure 3d‐ii). Considering the original emission intensity difference and the area‐induced visual difference, the ratio of the averaged emission in the whole area after washing to that before washing was analyzed (Figure 3e). The PEI_600_‐UCNPs emission showed a much higher retention capability: 53‐fold higher than that of the OA‐UCNPs and 4.8‐fold higher than that of the ligand‐free UCNPs (Figure S6, Supporting Information), indicating that the surface modification of UCNPs with appropriate molecular weight PEI can effectively enhance marking efficiency on the skin. In addition, the marking properties were analyzed with regard to the amount of surface PEI; it was determined that a molar ratio of 1:5 for UCNPs and PEI_600_ is especially favorable to achieve the best marking performance (Figure S7, Supporting Information).

To further verify whether the marking performance was dominantly facilitated by penetration, emission in the cross‐sectional images of marked porcine skin was compared with that of the original morphology (Figure 3f). It is apparent that the dominant emission comes from marked UCNPs on the skin surface for all samples. However, considering that these surface‐adhered UCNPs are easily removed by washing, emission inside the skin tissue should be given attention. Compared to the invisible internal emission for the PEI_50000_‐UCNP‐marked skin, all the other PEI‐UCNPs can penetrate the skin tissue. The penetration depth increased rapidly from 22.0 to 125.6 µm with a decrease in the molecular weight of PEI from 50 000 to 600 (Figure 3g). By extending the residence time of PEI_600_‐UCNPs on the skin from 2 to 12 h, the penetration depth could be increased to a value as large as 144.6 µm, indicating that the marking period could be greatly elongated if the marked area is not promptly cleared. It should be noted that PEI‐UCNPs have been proven to have low cytotoxicity in epidermal tissue engineering constructs^[^ ^23b^ ^]^ and PEI‐UCNPs have clearance and excretion capabilities after their intravenous, intraperitoneal, and intragastric administration.^[^ ^38^ ^]^ Thus, even if a small amount of PEI‐UCNPs could cause consistent permeation of the skin, they still could be metabolized. Furthermore, based on the results from the standard 3‐(4,5‐dimethylthiazol‐2‐yl)‐2,5‐diphenyltetrazolium bromide assay (Table S2, Supporting Information), at the concentration of the taggant solution we generally used the amount of the penetrated PEI_600_‐UCNPs into the epidermis is far below 100 µg mL^–1^, indicating that the present PEI_600_‐UCNPs are safe to be used as skin taggant.

**Figure 4:**
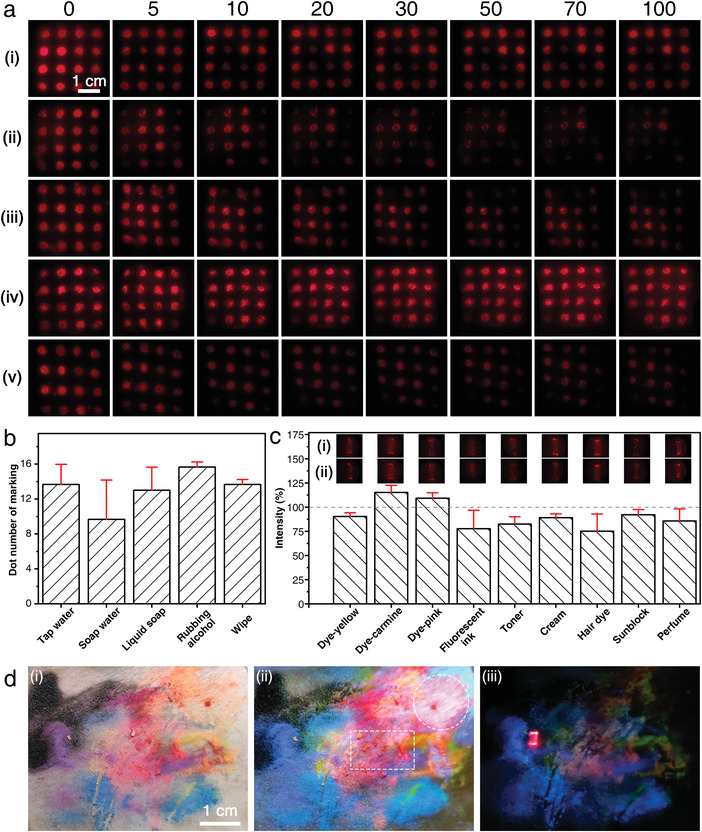
a) Luminescent images of PEI_600_‐UCNP marked in a 4 × 4 array on porcine skin before and after washing with different cycles (5–100) under 980 nm excitation: i) tap water flushing, ii) soap water washing, iii) liquid soap washing, iv) rubbing alcohol spraying, and v) wet tissue wiping. b) Number of dots after washing 100 cycles. c) Anti‐interference performance evaluation of the PEI_600_‐UCNPs marking on porcine skin comparing emission intensity i) before and ii) after shading of fluorescent dyes or cosmetics as well as the corresponding luminescent images. d) The comparison of the discrimination of the PEI_600_‐UCNPs marked area on porcine skin shading with various daily interferents under i) natural light, ii) 365 nm, and iii) 980 nm illumination. Data are shown as mean ± s.d. (*n* = 3).

To further evaluate the skin marking performance of the PEI_600_‐UCNPs directly, porcine skin marked with a 4 × 4 dot array of PEI_600_‐UCNPs was washed with different methods, such as vigorous tap water flushing, soap water washing, liquid soap washing, rubbing alcohol spraying, and wet tissue wiping (**Figure** **4**a), which are in accordance with the optimal wash solutions identified by the World Health Organization.^[^ ^39^ ^]^ The original luminescent arrays show bright red emissions with high contrast to the unmarked areas. With the increase of the washing cycles, although the entire emission tends to get weak, tap water flushing and rubbing alcohol spraying seem to have no obvious influence on observed brightness. The liquid soap washing and wet tissue wiping methods effectively removed the taggant when consistently increasing the washing cycles, while soap water washing is most effective at removal. However, no matter how robust the washing method is, most of the dots in the array can still be distinguished by simply improving the ISO of the mobile phone (Figure S8, Supporting Information).

Analysis of the washing effects indicates that the average emission signal decreases rapidly in the first ten cycles, as the skin‐adhered taggants are vulnerable to being washed away; in contrast, taggants that penetrate the stratum corneum and form strong electrostatic interactions with skin are more difficult to remove, and thus the signal tends to stabilize subsequently (Figures S9 and S10, Supporting Information). The retention of the luminescent array can be visualized by counting the number of dots remaining. At least ten dots could be maintained after 100 washing cycles (Figure 4b), retaining the original array characteristic well and confirming that effective electrostatic interaction between PEI and skin induced chemical adhesion and penetration to enhance its marking performance. In addition, the long‐term marking performance of the taggant was further verified as PEI_600_‐UCNPs displayed superior temperature adaptability by maintaining a distinct emission pattern at −20, 20, and 40 °C for as long as 69 days in the laboratory (Figure S11, Supporting Information).

To evaluate the anti‐interference properties of the PEI_600_‐UCNPs as a taggant in practical scenarios, three kinds of fluorescent dyes, fluorescent ink, personal care products and cosmetics, and hair dye were selected as the interferents (Figure 4c and Figure S12, Supporting Information). The intensity of the characteristic red emission exhibited only a limited decrease and remained above 82.4% in the presence of these interferents, except in the presence of fluorescent ink and hair dye, for which the intensity decreased to 75% relative to the uncontaminated taggant fluorescence. It is considered that viscous substances like pure fluorescent ink and hair dye may physically shield against red emission. These interferents would not cause an obvious change in brightness in the luminescent image observed simply by the naked eye (insets in Figure 4c).

In addition, considering complex practical scenarios in which various colorful or fluorescent substances are present, it is inevitable that these interferents will also be gathered on the skin. To precisely simulate this situation, the porcine skin was marked with a mixture consisting of seven fluorescent dyes with different long afterglow emissions: red fluorescent CdSe@ZnS QDs and a fluorescent ink, personal care products and cosmetics (toner, cream, sunblock, perfume), hair dye, and PEI_600_‐UCNPs. A colorful image with numerous red, blue, black, purple, orange, yellow, and green tones was observed under natural light (Figure 4d‐i), whereas areas marked with only CdSe@ZnS QDs or PEI_600_‐UCNPs are invisible. Upon 365 nm light irradiation, the upper right area marked with dash‐line circle exhibits red emission from CdSe@ZnS QDs, whereas it is difficult to distinguish this characteristic emission from the other red fluorescent dyes in the dash‐line rectangle area (Figure 4d‐ii). In contrast, the rectangle displaying bright red emission from PEI_600_‐UCNPs under 980 nm irradiation can be easily distinguished from all the background colors or emissions (Figure 4d‐iii), clearly demonstrating the superior performance of PEI_600_‐UCNPs as a covert taggant with anti‐interference properties even in extremely complex samples.

**Figure 5:**
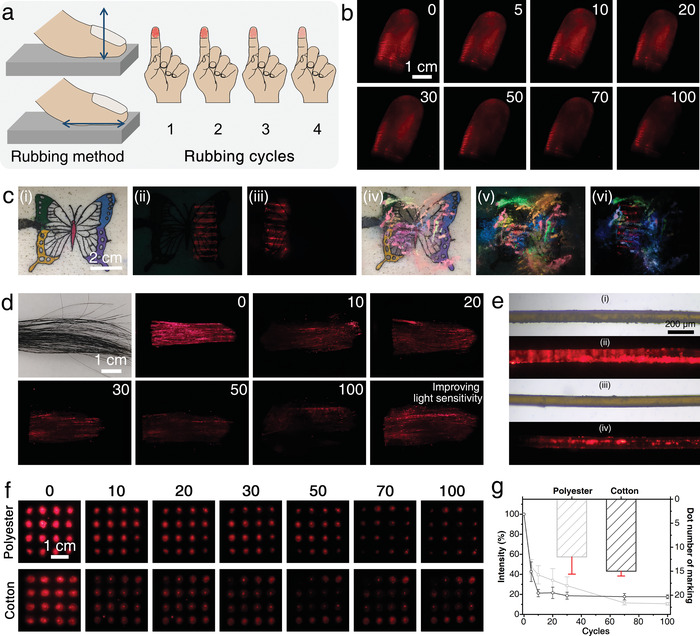
a) Schematic and b) images of fingerprint visualization by PEI_600_‐UCNPs markings after multiple rubbing cycles under 980 nm excitation. c) Images of tattooed porcine skin marked by PEI_600_‐UCNPs under i) natural light, ii) 365 nm, and iii) 980 nm illumination, and iv–vi) with the further addition of various fluorescent dyes. d) The optical and luminescent images of a bundle of hair marked with PEI_600_‐UCNPs washed with different cycles (0–100). e) The microscopic images of an individual hair i,ii) marked with PEI_600_‐UCNPs and iii,iv) after washing with 100 cycles under natural light and 980 nm illumination. f) The luminescent changing images of PEI_600_‐UCNP‐marked cloth, including polyester and cotton, after washing with different cycles (10–100). g) Luminescent intensity ratio and number of dots remaining on the cloth after washing 100 cycles. Data are shown as mean ± s.d. (*n* = 3).

To further prove the applicability of PEI_600_‐UCNPs as a skin taggant, its practical performance with fingerprints should be considered, since touch contacts could result in unnecessary transfer and loss of the taggant, and thus decrease the effectiveness of visualization and identification. An initial fingerprint marking was achieved by pressing a finger into a solution of PEI_600_‐UCNPs on a flat plate. Fingerprint images were acquired by rubbing the marked finger on stainless steel tables, walls, or wood randomly for different cycles; images were also directly captured on the finger (**Figure** **5**a). Clearly defined and bright luminescence images allowed fingerprint ridge pattern details to be easily recognized even after 30 cycles of rubbing (Figure 5b). Thereafter, although the visualized fingerprint gradually disintegrates with increasing rubbing cycles, compared to the bright edge which most probably retains due to the limited contact, the characteristic red luminescent residue still can be observed in the middle of the fingerprint after 100 cycles rubbing, indicating superior marking performance owing to the effective penetration of the taggant in the skin.

A butterfly tattoo marked on its right wing with the CdSe@ZnS QDs bar code and left wing with the PEI_600_‐UCNPs bar code was designed to evaluate the anti‐interference capability of PEI_600_‐UCNPs. The butterfly tattoo is multicolored and the taggant is invisible under natural light (Figure 5c‐i). Once illuminated by UV or NIR light, CdSe@ZnS QDs or PEI_600_‐UCNPs immediately emit characteristic red luminescence without interference from multiple tattoo pigments (Figures 5c‐ii,iii). Considering a more complex fluorescence environment, e.g., fluorescent night running, various fluorescent dyes with different long afterglow emissions were marked on the tattooed skin (Figure 5c‐iv). The characteristic red fluorescence of CdSe@ZnS QDs is almost impossible to observe (Figure 5c‐v), while the upconversion bar code for PEI_600_‐UCNPs is clearly visible under 980 nm illumination (Figure 5c‐vi), demonstrating the superiority of the PEI_600_‐UCNPs as a taggant for anti‐interference skin marking even with specifically intended concealing.

Moreover, the PEI_600_‐UCNP taggant is also efficient in hair marking after repeated shampoo washings (Figure 5d). This good marking effect can be attributed to the thorough penetration of PEI_600_‐UCNPs into hair, since the radius of a single hair is within 83 µm, which is much less than the penetration depth in skin of 144.6 µm (Figure 5e). Considering that there is less exposed skin, especially in cold winter, cloth marking could be another choice to realize individual marking, and thus cloth materials, including polyester and cotton, were marked with the PEI_600_‐UCNPs aqueous solution by forming a 4 × 4 luminescent dot array (Figure 5f). The red luminescent pattern was well maintained even after 100 cycles of washing regardless of the specific cloth materials. After ten cycles of washing, 80% of the luminescence would be lost (Figure 5g), owing to the peeling off of the adhered taggants. The ≈20% residue inside the fiber is capable of maintaining the marked array with 12–15 dots, which is sufficient to retain the original marking information.

## 3 Conclusion

In conclusion, we have demonstrated a new approach for individual marking that couples adhesion between PEI amino groups and the intrinsic functional groups on the skin, with the chemical penetration of PEI‐functionalized Mn^2+^‐doped NaYF_4_:Yb/Er UCNPs into the skin, thus endowing the taggant with a highly retentive, anti‐interference, and covert marking efficiency. A key feature of the PEI‐UCNP taggant is that the electrostatic interaction between PEI and lipid enhances its chemical penetration and improves retention of the luminescent tag. PEI_600_‐UCNPs demonstrated improved marking efficiency: a 53‐fold enhancement of luminescence retention was observed for PEI_600_‐UCNPs compared to OA‐UCNPs after robust scrubbing. In addition, the upconversion design prioritizes covertness and anti‐interference in distinguishing the taggant from a colorful and fluorescent background, even under extremely complex circumstances. Furthermore, the applicability of the proposed PEI_600_‐UCNP taggant for complex mixtures was demonstrated in an intricate tattooed skin marking in the presence of various fluorescent dyes and QDs, as well as in fingerprint, hair, and cloth marking applications. PEI‐functionalized UCNPs are promising for highly retentive, anti‐interference, and covert individual marking applications. While further investigations, such as the study of PEI‐UCNP metabolism inside skin, are required, PEI‐functionalized UCNPs have great potential for special character tracking in real cases.

## 4 Experimental Section

### Materials

MnCl_2_·4H_2_O (99.99%), Y(NO_3_)_3_·6H_2_O (99.99%), Yb(NO_3_)_3_·5H_2_O (99.99%), Er(NO_3_)_3_·6H_2_O (99.99%), oleic acid (OA, AR), ethanol (AR), cyclohexane (AR), sodium hydroxide (NaOH, AR), sodium fluoride (NaF, AR), and dimethyl sulfoxide (DMSO, AR) were all obtained by Aladdin (Shanghai, China) and used without further purification. Branched polyethyleneimine (molecular weight: 600, 1800, 3500, 10 000, 50 000) was of analytical grade and purchased from Xiya Chemical Technology Co., Ltd. (Shandong, China). Phosphate‐buffered saline (PBS) buffer was purchased from Beijing Dingguo Changsheng Biotechnology Co., Ltd. (Beijing, China). Ultrapure deionized water (18 MΩ) was prepared by laboratory ultrapure water machine (Water purifier, WP‐UP‐2). The artificial membranes (namely, Strat‐M membranes) were obtained from Shanghai Tuling Trading Co., Ltd. (Shanghai, China). The fluorescent ink was purchased from Zhengzhou Wanyintong Seal Technology Co., Ltd. (Henan, China). The fluorescent dyes composed of a basic formula of 45% alumina, 54% strontium carbonate, 0.5% europium sesquioxide, and 0.5% dysprosium oxide with different long afterglow emissions were bought from Nanjing Juen Technology Co., Ltd. (Jiangsu, China). Commercial Kiehl's calendula herbal extract alcohol‐free toner, Clinique oil‐free gel, Schwarzkopf natural & easy hair dye with 3.0/9 dark brown, Anessa sunblock with an SPF 50+ and PA+++++, Victoria's secret fragrance mist brume perfume were obtained from the local shopping center. Cotton swabs, clothes (cotton, polyester) were commercially purchased from HOTWIND Co., Ltd. (Shanghai, China). CdSe@ZnS QDs were synthesized based on a literature protocol.^[^ ^40^ ^]^

### Characterizations

XRD measurement was conducted using powder XRD (Bruker D8 Advance, with Cu‐K_*α*_ radiation operating at 40 kV and 40 mA, scanning from 20° to 80°). Field‐emission scanning electron microscope (JEOL JSM‐7610F Plus) and TEM (JEOL JEM‐F200, 200 kV) equipped with an energy‐dispersive X‐ray spectrometer were used to characterize the morphology and composition of the samples. XPS (K‐Alpha+), with a twin‐anode Al K_*α*_ (1486.6 eV) X‐ray source, was used to quantitatively evaluate the composition of the samples. FT‐IR spectra were obtained using a PerkinElmer Frontier FT‐IR spectrometer with a Smart Orbit diamond crystal attenuated total reflectance attachment. Zeta potential analyzer (Zetasizer Nano ZS90) was used to characterize the Zeta potential. Thermogravimetric analysis measurements were performed in air up to 800 °C at a heating rate of 10 °C min^−1^ using a Simultaneous Thermal Analyzer (STA 8000). Upconversion luminescence spectra were measured on a grating spectrometer (Ocean Optics, Maya 2000 Pro) by a 980 nm laser (CNIlaser, FC‐980‐3 W, 210602‐R447684) excitation in a dark room. The digital photographs were captured by a Vision Datum Mars 5000S‐20gc industrial camera under 980 nm excitation (CNIlaser, PGL‐VI‐980‐400 mW, CA11845‐2) or Xiaomi Redmi K30 smartphone under 980 nm excitation (CNIlaser, FC‐980‐30 W, CI90239) at a power density of 1.2 W cm^–2^ for porcine skin and cloth, and 0.6 W cm^–2^ for finger and hair. The optical micrographs and dark‐field images were performed on Nikon Ti‐E inverted fluorescence microscope. The *R* value before or after washing was extracted using the ImageJ software. The dot array was prepared by dropping 40 µL of PEI_600_‐UCNPs solution (2 mg mL^–1^) into each hole of a 3D‐printed resin mold with 16 holes (0.4 × 0.4 cm^2^).

### Synthesis of the Mn^2+^‐Doped NaYF_4_:Yb/Er Upconversion Nanoparticles (OA‐UCNPs)

In a typical experiment,^[^ ^41^ ^]^ NaYF_4_:Yb/Er nanoparticles doped with Mn^2+^ ions were prepared by changing the value of *x* in the reaction system (“*x*” is the volume of 0.5 m Mn^2+^ ion solution, 0 ≤ *x* ≤ 3.2). In a typical synthesis route, first, 0.6 g sodium hydroxide and 3 mL deionized water were mixed to form a clear and transparent solution, followed by adding 10 mL OA and 20 mL ethanol. *X* mL of 0.5 m MnCl_2_, (3.2−*x*) mL of 0.5 m Y(NO_3_)_3_, 1.8 mL of 0.2 m Yb(NO_3_)_3_, and 0.2 mL of 0.2 m Er(NO_3_)_3_ were added to a mixture by vigorous agitation for 30 min. Then, 2 × 10^−3^ m NaF was then slowly added into the flask. After vigorous stirring at room temperature for 20 min, the colloidal solution was transferred into a 50 mL Teflon‐lined autoclave, sealed and heated at 200 °C for 8 h. The systems were then allowed to naturally cool to room temperature, and thereafter the obtained products were washed with ethanol and cyclohexane, and then froze drying.

### Synthesis of the PEI Functionalized UCNPs (PEI‐UCNPs)

In a typical experiment,^[^ ^42^ ^]^ 90 mg OA‐UCNPs and 43 mL DMSO were added to a 100 mL round‐bottom flask with magnetic stirring to form a transparent solution. Then mixed with 450 mg PEI_10000_ in 2.7 mL of DMSO by ultrasonication, the reaction mixture was kept refluxing at 95 °C until the solution turned light yellow. The resulting solution was cooled to room temperature. The final product was collected by centrifugation, washed several times with ethanol and water, and then froze drying. A set of PEI with different molecular weight (from 600 to 50 000) and different surface PEI amounts (a molar ratio of UCNPs/PEI from 1:1 to 1:20) grafted UCNPs was prepared by using the same method, only the corresponding PEI was replaced.

### Calculation Section

The GROMACS 2021.4 package,^[^ ^43^ ^]^ Multiwfn software,^[^ ^44^ ^]^ VMD program,^[^ ^45^ ^]^ and ATB^[^ ^46^ ^]^ website (version 3.0) were used in simulation of permeation section.

Synthesis of ligand‐free UCNPs, porcine skin samples, evaluation of the marking performance of series of UCNPs on porcine skin, measurements of skin permeation, evaluation of PEI_600_‐UCNPs cytotoxicity in vitro, evaluation of the skin marking performance of the PEI_600_‐UCNPs by different washing method, evaluation of the long‐term marking performance of PEI_600_‐UCNPs, evaluation of the anti‐interference performance of the PEI_600_‐UCNPs, evaluation of practical marking performance on fingerprints, evaluation of practical marking performance on hair, evaluation of practical marking performance on cloth, the surface of NaYF_4_ and absorption models, as well as MD simulation of permeation, are all placed in the Supporting Information.

## Conflict of Interest

The authors declare no conflict of interest.

## Supporting information

Supporting Information

## Data Availability

The data that support the findings of this study are available in the Supporting Information of this article.
