# Supplementary material for: Highly Retentive, Anti‐Interference, and Covert Individual Marking Taggant with Exceptional Skin Penetration
Source: Adv Sci (Weinh). 2022 Jun 24;9(25):2201497. doi: 10.1002/advs.202201497 (PMC9443463; doi:10.1002/advs.202201497)
Supplement: Supplementary file 1 — Supporting Information [file ADVS-9-2201497-s001.pdf]

ADVANCED SCIENCE
Open Access

Supporting Information

for *Adv. Sci.*, DOI 10.1002/advs.202201497

Highly Retentive, Anti-Interference, and Covert Individual Marking Taggant with Exceptional Skin Penetration

Lianggen Zhong, Jiguang Li, Baiyi Zu, Xiaodan Zhu, Da Lei, Guangfa Wang, Xiaoyun Hu, Tianshi Zhang and Xincun Dou**

Supporting Information

Highly Retentive, Anti-interference, and Covert Individual Marking Taggant with Exceptional Skin Penetration

Lianggen Zhong,# Jiguang Li,# Baiyi Zu, Xiaodan Zhu, Da Lei, Guangfa Wang, Xiaoyun Hu, Tianshi Zhang, and Xincun Dou**

Experimental Section

Synthesis of ligand-free UCNPs[1]: OA-UCNPs were dispersed in 10 mL cyclohexane solution, followed by adding 10 mL deionized water. The reaction was performed with stirring for 1 h at 45 °C while maintaining the pH at 4 by adding a solution of HCl 0.1 M. Removing supernatant and adding acetone to precipitate, the UCNPs of the aqueous solution were collected *via* centrifugation at 12000 rpm for 30 min, washed several times with ethanol and deionized water, and then froze drying.

Porcine skin samples: Fresh porcine skin was obtained from a local supermarket and the hair was carefully removed by a trimmer to ensure skin was not damaged. The skin was frozen at -20 °C.[2] Before use, the frozen skin was thawed on ice, cut into small pieces and washed with PBS buffer. The rubber O-rings (I.D. = 4.5 mm; W = 1.78 mm) were clamped and sealed with vaseline on the top of each piece of skin.[3] The skin pieces were then placed in a 6-well microplate and PBS buffer (600 μL per well) was added in order to immerse the dermis and leave the epidermis at the air-medium interface.

Evaluation of the marking performance of series of UCNPs on porcine skin: Each O-ring on the porcine skin was filled with 20 μL 20 mg mL^{-1} series of UCNPs solution (*e.g.*, PEI-UCNPs with different molecular weights of PEI, PEI-UCNPs with different PEI amounts, OA-UCNPs and ligand-free UCNPs). The skin was then incubated with PBS buffer for 2 h in a chamber at 30 °C. Afterwards, the marked porcine skin was washed by the wet cotton swabs soaking with liquid soap solution for 30 s, and the corresponding luminescent images were captured by an industrial camera under 980 nm excitation before and after washing.

Measurements of skin permeation: Each O-ring was filled with 10 μL of PEI-UCNPs solution (80 mg mL^{-1}). The skin was then incubated for 2 h in a humidity chamber at 30 °C. Following 2 h incubation, the skin was washed with PBS buffer. Thereafter, they were cut into small pieces, approximately 5.0 × 5.0 mm^2, and placed in a cryomold (Tissue-Tek® Biopsy, square 10 × 10 × 5 mm^3), embedded in OCT (Leica), frozen at -80 °C. The specimens were sectioned perpendicular to the skin surface into 20 μm thick slices using a cryostat (Leica CM1950, Germany), and placed on anti-frost glass slides. The slides were imaged on an inverted fluorescence microscope microscopy.

Evaluation of PEI_{600}-UCNPs cytotoxicity in vitro: Immortalized human keratinocyte cell line, HaCaT, was applied to investigate the cytotoxicity of the PEI_{600}-UCNPs. HaCaT cells were seeded in a flat bottom 96-well plate with 100 μL per well, incubated for 12 h prior to media removal. Then, 20 μL of the sample was added to each well and the plates were incubated at 37 °C with 5% CO_2. After 48 h, the culture medium was discarded and 100 μL of 0.5 mg mL^{-1} 3-(4,5-dimethylthiazol-2-yl)-2,5-diphenyltetrazolium bromide (MTT) agent was added to each well. Under the same condition, the cell lines were incubated for 2 h, and optical density (OD) at 570 nm was measured by MB microplate reader.

Evaluation of the skin marking performance of the PEI_{600}-UCNPs by different washing method: The dot array marked on the porcine skin was prepared by dropping 40 μL of PEI_{600}-UCNPs solution (2 mg mL^{-1}) into each hole of a 3D-printed resin mold with 16 holes (0.4 × 0.4 cm^2). Then the porcine skin marked with PEI_{600}-UCNPs dots array was washed with different cycles (5-100). (i) Tap water flushing: the marked skin was washed with top water (v = 30 L s^{-1}, h = 39 cm) for 20 s as one cycle. (ii) Soap water washing: the marked skin was washed with one finger for circular movement in assist of soapy water (50 μL, 10 mg mL^{-1}) for 20 s as one cycle. (iii) Liquid soap washing: the marked skin was washed with one finger for circular movement in assist of liquid soap water (50 μL) for 20 s as one cycle. (iv) Rubbing alcohol spraying: the marked skin was sprayed with rubbing alcohol and each spraying represents one cycle. (v) Wet tissue wiping: the marked skin was wiped with a wet towel wipe which was added a 500 g weight on it, then drag the wet towel at a constant speed,

each wipe represents one cycle. To distinguish the marking dots in the array after washing 100 cycles, the ISO of the mobile phone was adjusted from 320 to 1600.

Evaluation of the long-term marking performance of PEI_{600}-UCNPs: Each O-ring was filled with 10 µL of PEI_{600}-UCNPs solution (2 mg mL^{-1}). The artificial skin was placed at -20, 20, 40 °C for a period of time, respectively, and the fluorescence spectra and images were measured and recorded.

Evaluation of the anti-interference capability of the PEI_{600}-UCNPs: Each O-ring was filled with 10 µL of PEI_{600}-UCNPs solution (20 mg mL^{-1}). After drying, the fluorescence spectra and the luminescent images of the PEI_{600}-UCNPs marking performances on porcine skin were measured and recorded under 980 nm excitation. The liquidous interferents including fluorescent dyes with long afterglow emissions (dye-yellow, dye-carmine and dye-pink, 1 mg mL^{-1}), toner, sunblock and perfume, were sprayed on the PEI_{600}-UCNPs marked porcine skin. The non-flowing interferents, fluorescent ink (1 mL), cream (2 mL), hair dye (1.5 mL), were firstly placed to the plastic wrap (14 × 14 cm^2). Then the plastic wrap was covered on the marked porcine skin with a gentle pressing. After the treatment with the interferents on the PEI_{600}-UCNPs marked porcine skin, the fluorescence spectra and the luminescent images were measured under 980 nm excitation.

The porcine skin marked with a mixture consisting of 7 fluorescent dyes with different long afterglow emissions, red-fluorescent CdSe@ZnS QDs and a fluorescent ink, some daily used skin care products, cosmetics (toner, cream, sunblock, perfume), hair dye as well as the PEI_{600}-UCNPs, was photographed under natural light, 365 nm and 980 nm excitation, respectively. Similarly, to simulate a more realistic scene, the porcine skin tattooed with "butterfly" pattern, the CdSe@ZnS QDs bar code and PEI_{600}-UCNPS bar code, was photographed under natural light, 365 nm and 980 nm excitation, respectively. Furthermore, 7 different fluorescent dyes with different long afterglow emissions were further sprayed on the tattooed porcine skin which was photographed under the same condition as above.

Evaluation of practical marking performance on fingerprints: An initial fingerprint marking was achieved by pressing the solution of PEI_{600}-UCNPs (2 mg mL^{-1}) on a flat plate.

Fingerprint images can be acquired by the marked finger rubbed on stainless steel tables, walls, wood randomly for different cycles and directly captured on finger.

Evaluation of practical marking performance on hair: 1 mL PEI_{600}-UCNPs solution (10 mg mL^{-1}) was dropped on a bundle of hair of the volunteers. After 2 h of drying, the bundle of hair was washed with shampoo solution (0.33 mg mL^{-1}) for 2 times and rinsed with water for 3 times, which was one cycle. The optical and luminescent images of a bundle of hair were recorded after washing with different cycles (0-100). Then an individual hair was selected and characterized by an inverted fluorescence microscope microscopy. To distinguish the marking hair after washing 100 cycles, the ISO of the mobile phone was adjusted from 1250 to 2500.

Evaluation of practical marking performance on cloth: The clothes (polyester, cotton) were cut into pieces (6 × 8 cm^2), the dot array marked on the cloth was prepared by dropping 40 µL of PEI_{600}-UCNPs solution (2 mg mL^{-1}) into each hole of a 3D-printed resin mold with 16 holes (0.4 × 0.4 cm^2). After drying, the cloth was washed by intense stirring (400 rpm) in the beaker in assist of washing powder solution (5.6 mg mL^{-1}), washing two minutes represents a cycle. The luminescent changing images of marked cloth were recorded before washing and after washing with different cycles (10-100).

Statistical Analysis: The images and fluorescence intensity were processed or analyzed by Image J. The size of UCNPs and average depth of skin penetration in the images were measured through Photoshop 2019C Software. All the results were reported as a mean with standard deviation using 2018 Origin Software. Sample size (n) of independent repeated experiments for each statistical analysis was given in the figure legends.

The surface of $NaYF_4$ and absorption models: In order to unravel the underlying mechanism of ligands adsorbed and bonded on the surface of $NaYF_4$, the density functional theory (DFT) based first principles calculations were performed to simulate the interactions between the surface of $NaYF_4$ (111) and selected ligands using CASTP[4] in Material Studio which implemented a Perdew Burke Ernzerhof (PBE) with a kinetic energy cut-off of 500 eV for the plane wave. The (111) surface structure was modeled using a super-cell of the bulk $NaYF_4$ (a = 6.001 Å, c = 3.603 Å), which consisted of a 5-layer slab separated by a vacuum of 20 Å. Because the long carbon chain is a sufficient distance from the active functional group

of OA and PEI ligands to make the surface binding energy negligible, two simplified molecules, namely $C_6H_{13}O_2$ and $C_4H_{12}N_2$ were conducted to mimic the adsorption characters of the OA and PEI ligands to the $NaYF_4$ facets, respectively. Moreover, only the interaction between the molecule and Y^{3+} ions was considered since the Y^{3+} ions in cubic $NaYF_4$ is preferential to bind with other groups. The energy of initial adsorption configurations of ligands onto the (111) surface was considered, and are illustrated in Table S1, respectively. The binding energy was determined by

$$E_b = E_{(111)/ligand} - [E_{(111)} + E_{ligand}]$$

Where E_b, $E_{(111)/ligand}$, $E_{(111)}$ and E_{ligand} represented the energies of the binding complex, the $NaYF_4$ (111) slab and the isolated molecule of ligand, respectively.

Molecular dynamics simulation of permeation: All molecular dynamics (MD) simulations were performed using the GROMACS 2021.4 package.[5] The GROMOS96 54A7[6] parameters obtained from ATB[7] website (version 3.0) were used to describe bonded and nonbonded interactions for all studied molecules. Water molecules were modelled with the SPC potential. Restrained electrostatic potential (RESP)[8] charges calculated at the B3LYP[9]/6-311G(d,p)[10] level were used to describe electrostatic properties of all molecules by using Gaussian[11] and Multiwfn[12] software. Electrostatic potential (ESP) was calculated at the same level by Multiwfn software. All isosurface maps and MD trajectory were rendered by VMD[13] program. In order to mimic a realistic skin layer, a simulation model was constructed *via* considering an equimolar ratio of the three most abundant skin lipids, namely ceramides (CER), free fatty acids (FFA) and cholesterol (CHOL). A large equimolar bilayer structure with a simulation box of dimensions of 8 nm × 8 nm × 11.5 nm was constructed using the individual components (Figure S5) of the skin layer lipids. Then the system was saturated with water, which includes 318 lipid molecules (106 CER, 106 CHOL and 106 FFA), 6 PEI and 10400 water molecules. For this system, the energy minimization was firstly performed by the steepest descent algorithm followed by 10 ns NPT (P = 1.01325 kpa and T = 310 K) simulation with a Velocity-rescale thermostat[14] and Berendsen barostat[15] with the time constants of couplings being 0.2 and 2.0 ps, respectively. Due to the weak coupling

between lipid bilayer and between lipid layer and water, false relative sliding is easy to occur, so translation is eliminated for upper and lower lipid layer and water respectively. Long-range electrostatic interactions were handled by the Particle-mesh Ewald (PME)[16] method with a cut-off of 1.0 nm used (for electrostatic and Lennard-Jones interactions). Finally, 650 ns NPT simulation with a Velocity-rescale thermostat[14] and Parrinello-Rahman barostat[17] (Semiisotropic is used to control the pressure so that the barostat is coupled separately in the XYZ direction) with the time constants of couplings being 0.2 and 2.0 ps of production of MD simulation was performed to obtain the final equilibrated. When calculating the interaction between molecules, the electrostatic and Lennard-Jones interactions was calculated by cut-off of 3.5 nm method.

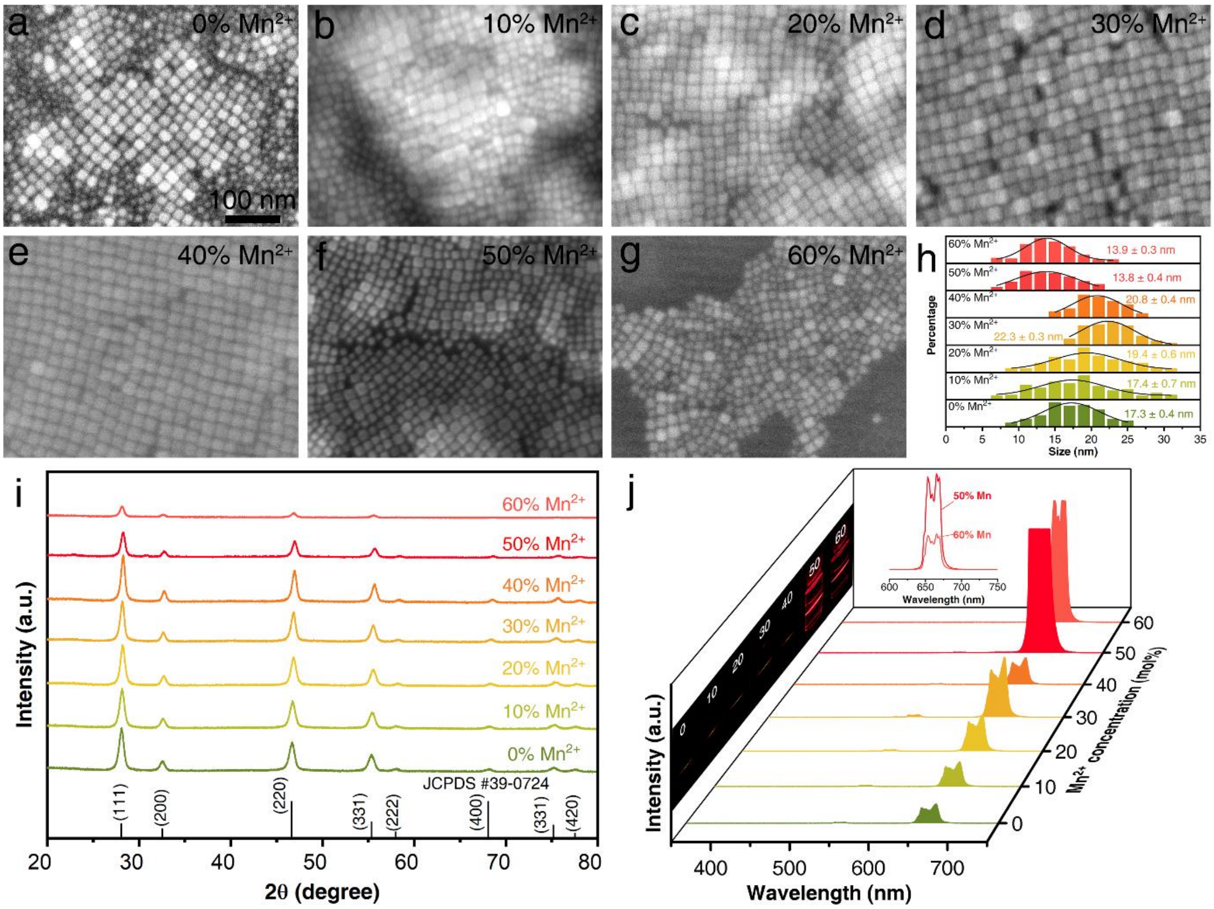

Figure S1. $NaYF_4$: Yb/Er UCNPs doped with different concentrations of Mn^{2+} with a molar ratio from 0 to 60% in the precursor comparing to the total Y amount including Yb and Er. (a-g) SEM images. (h) Size distribution histograms. Data are shown as mean ± s.d. (n=100). (i) XRD patterns. (j) Upconversion emission spectra and digital photographs.

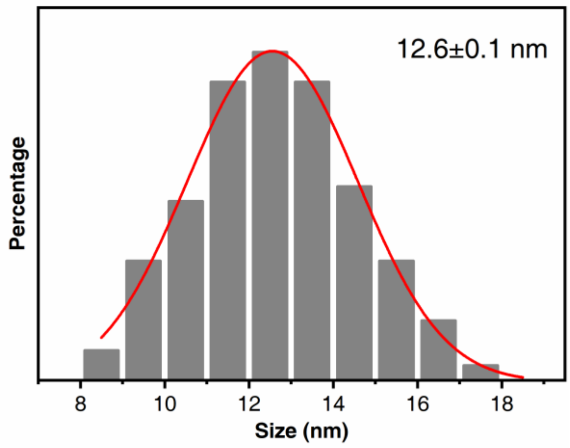

Figure S2. Size distribution histogram of OA-UCNPs. Data are shown as mean ± s.d. (n=100).

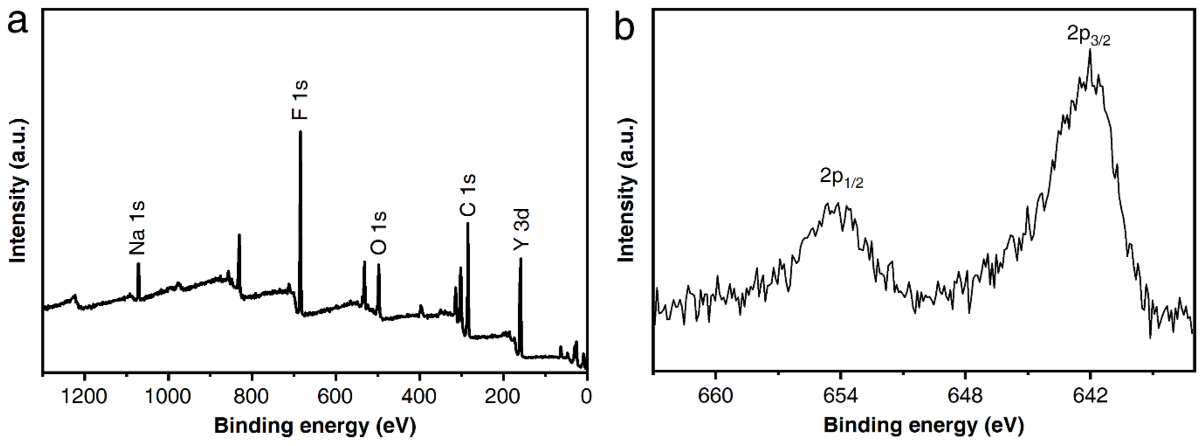

Figure S3. XPS spectra. (a) $NaYF_4$: Yb/Er UCNPs without Mn^{2+}. (b) Mn 2p of the optimized Mn^{2+}-doped $NaYF_4$: Yb/Er UCNPs.

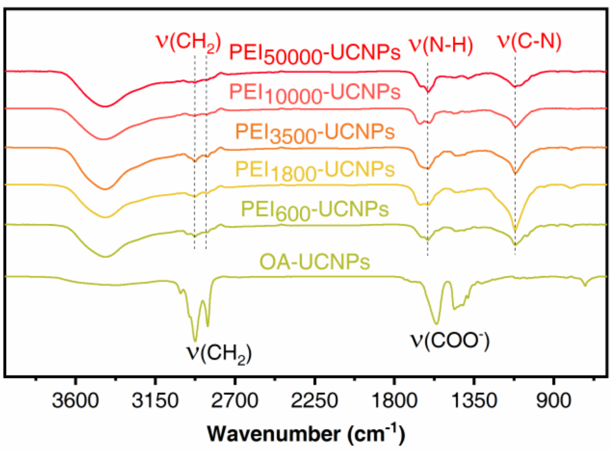

Figure S4. FT-IR spectra of OA-UCNPs and PEI-UCNPs.

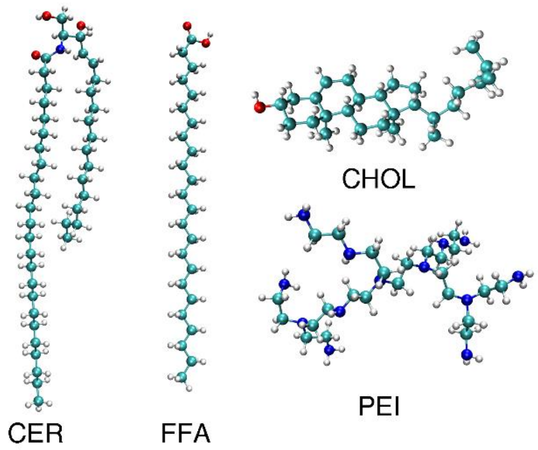

Figure S5. The 3D molecular structures of each component (CER, CHOL, and FFA) of the stratum corneum lipid model and PEI. Atomic color code: O (red); N (blue); C (cyan); H (grey).

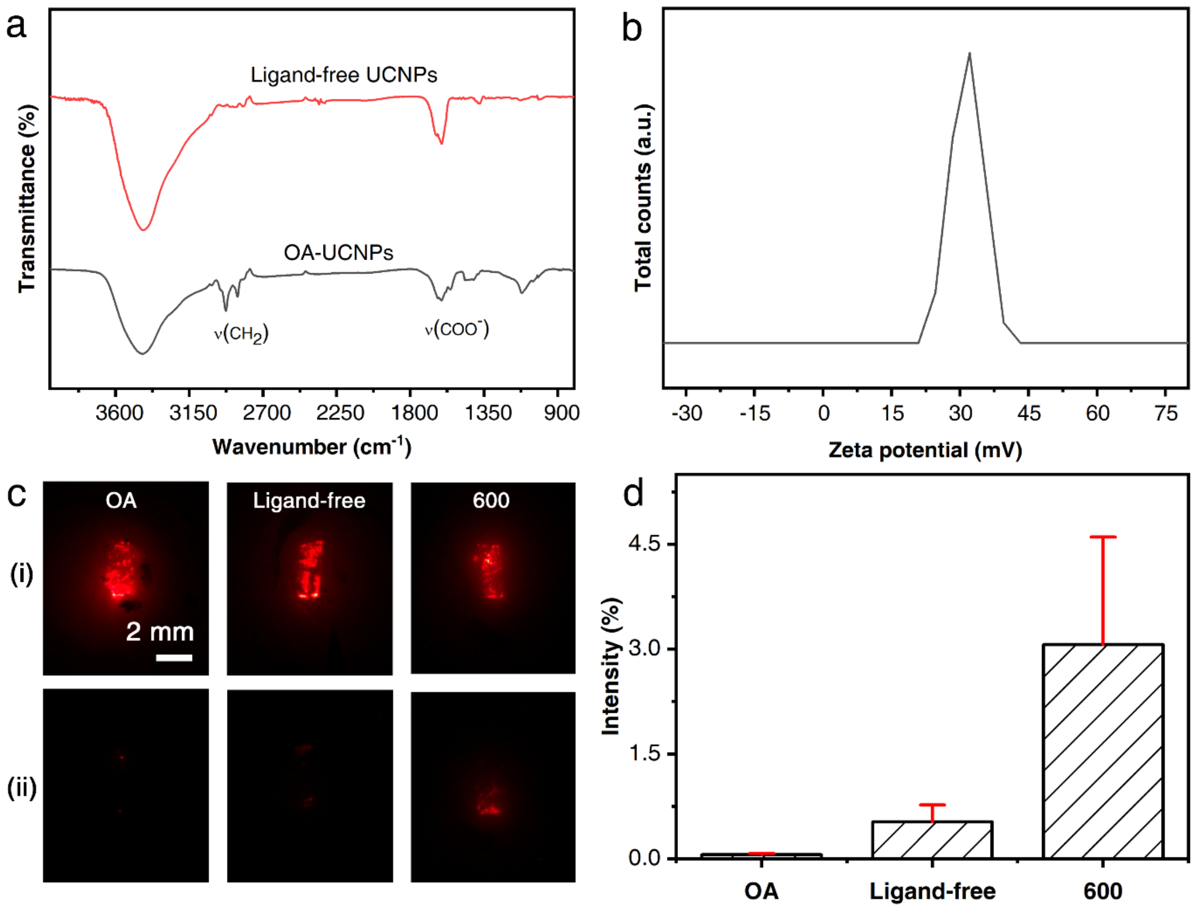

Figure S6. (a) FT-IR spectra of OA-UCNPs, ligand-free UCNPs. (b) Zeta potential of ligand-free UCNPs. (c) Comparison of the luminescent images of OA-UCNPs, ligand-free UCNPs and the PEI_{600}-UCNPs marking on porcine skin (i) before and (ii) after washing under 980 nm excitation. (d) The corresponding luminescence retention ratios after washing. Data are shown as mean ± s.d. (n=3).

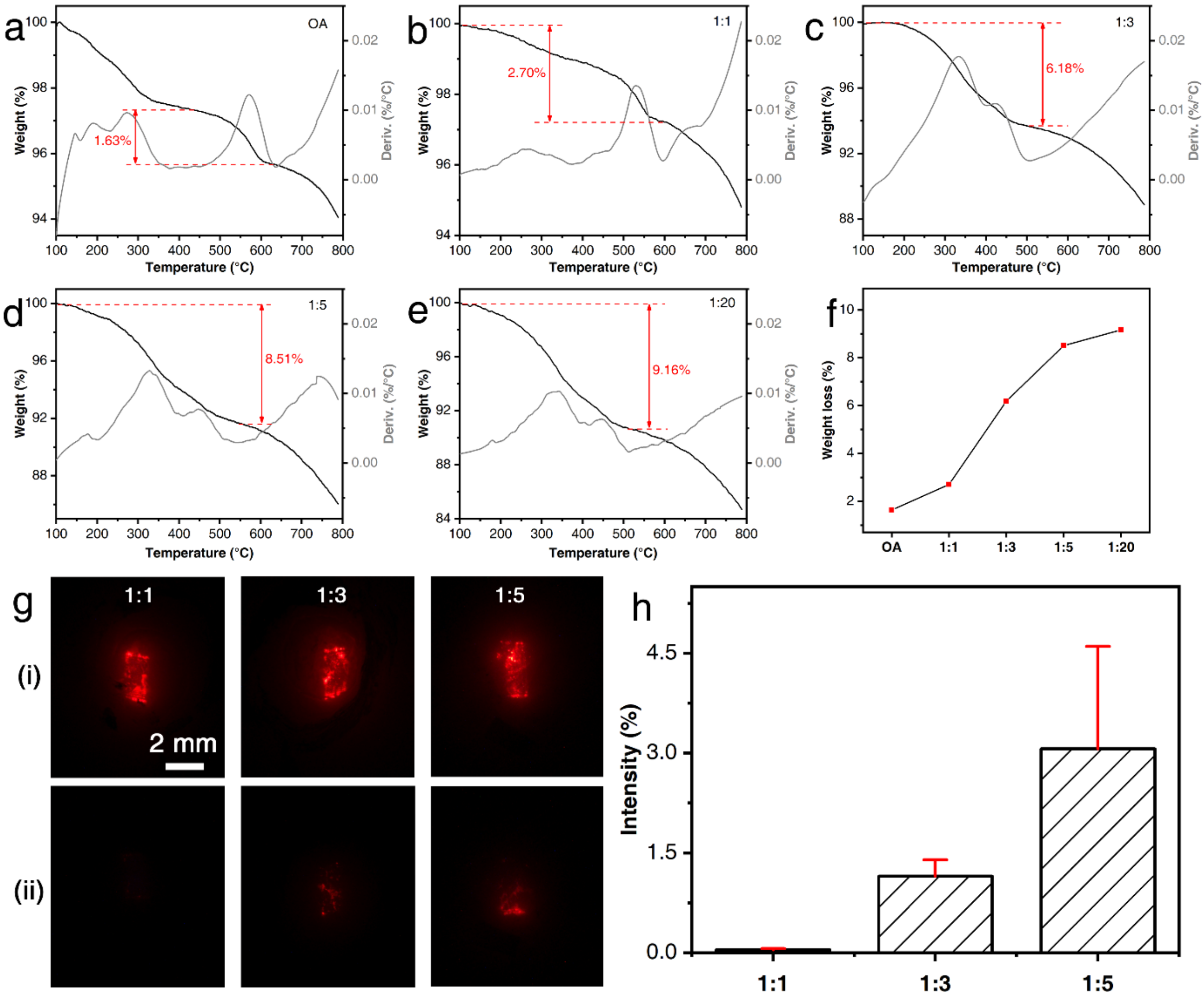

Figure S7. Thermogravimetric curves for (a) OA-UCNPs and (b–e) the PEI_{600}-UCNPs with a molar ratio of UCNPs/PEI from 1:1 to 1:20. (f) The corresponding mass loss. (g) Comparison of the luminescent images of the PEI_{600}-UCNPs with different molar ratio marking on porcine skin (i) before and (ii) after washing under 980 nm excitation. (h) The corresponding luminescence retention ratios after washing. Data are shown as mean ± s.d. (n=3).

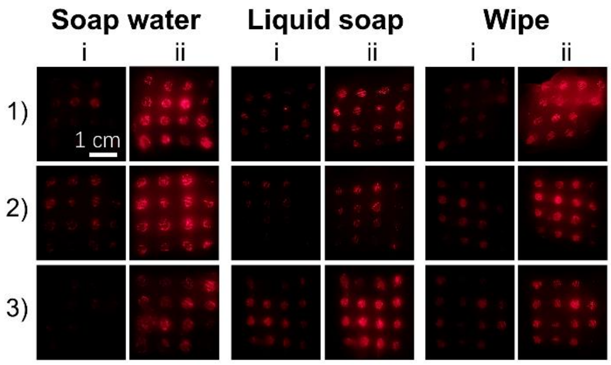

Figure S8. The porcine skin after washing 100 cycles with soap water, liquid water and wipe (i) the pristine photos and (ii) improving the ISO of the the mobile phone. 1–3: Three repetitions.

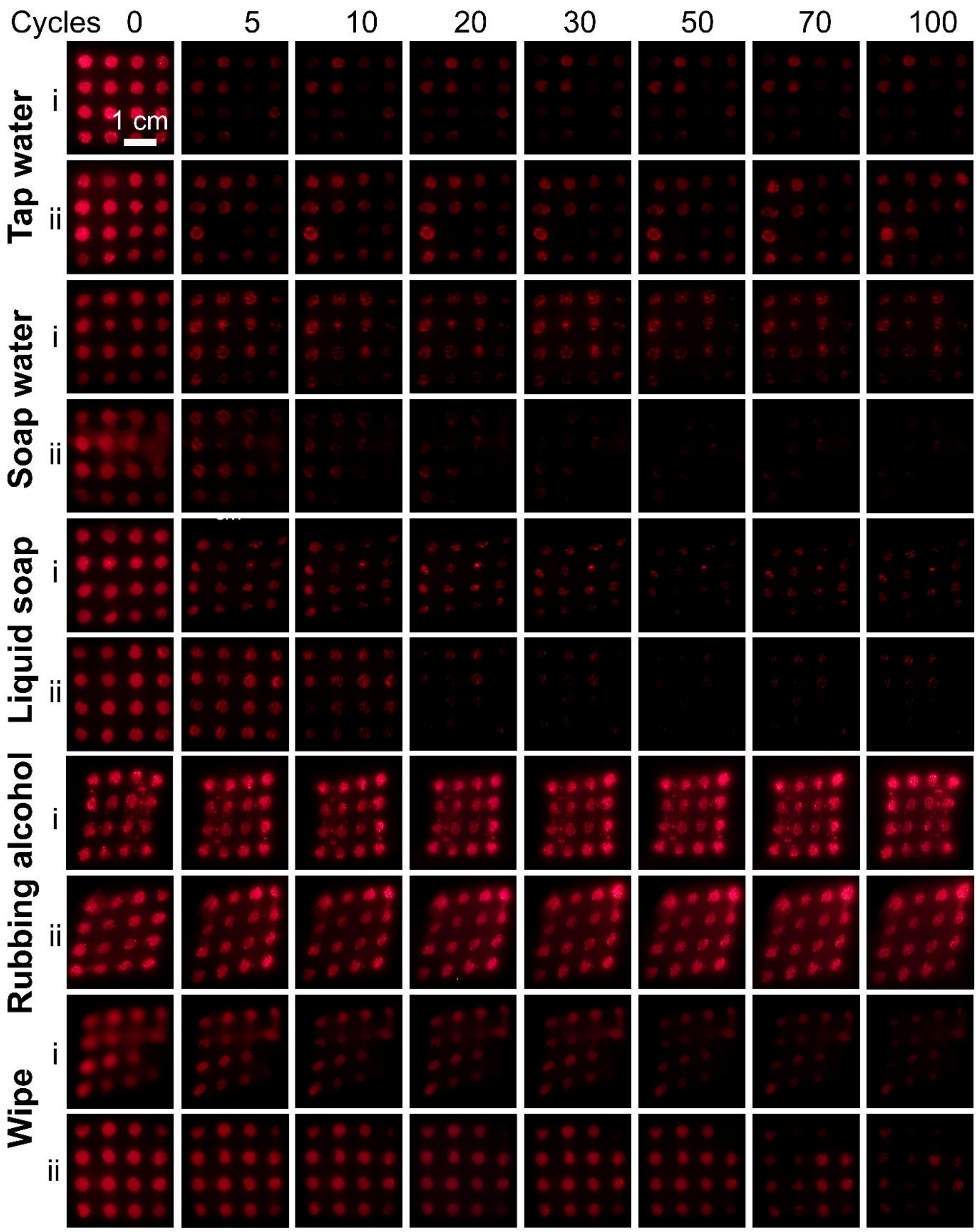

Figure S9. The luminescent images of the PEI_{600}-UCNPs marked on porcine skin before and after washing with different cycles and methods under 980 nm excitation.

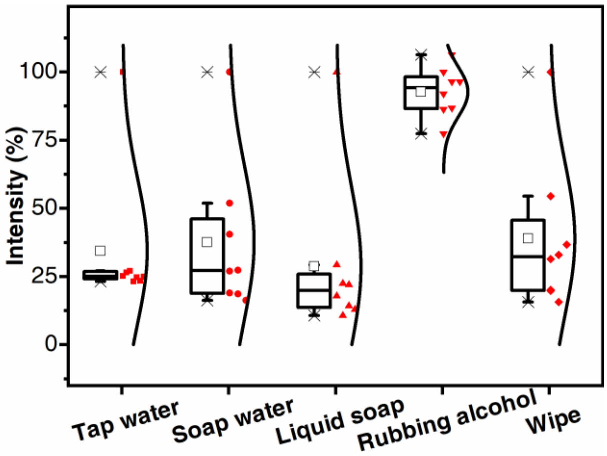

Figure S10. Luminescent intensity ratio of PEI_{600}-UCNPs on the skin after washing 0, 5, 10, 20, 30, 50, 70, 100 cycles.

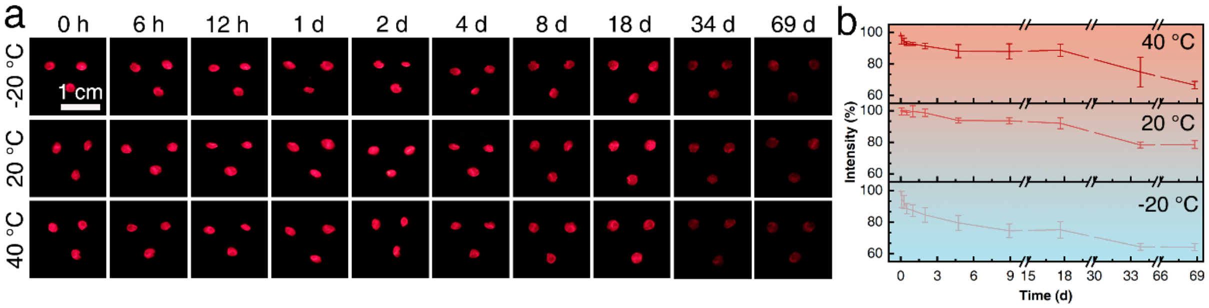

Figure S11. Temperature adaptability of PEI_{600}-UCNPs at different temperatures of -20, 20, 40 °C on artificial membranes. (a) Dark field images. (b) Luminescent performance. Data are shown as mean ± s.d. (n=3).

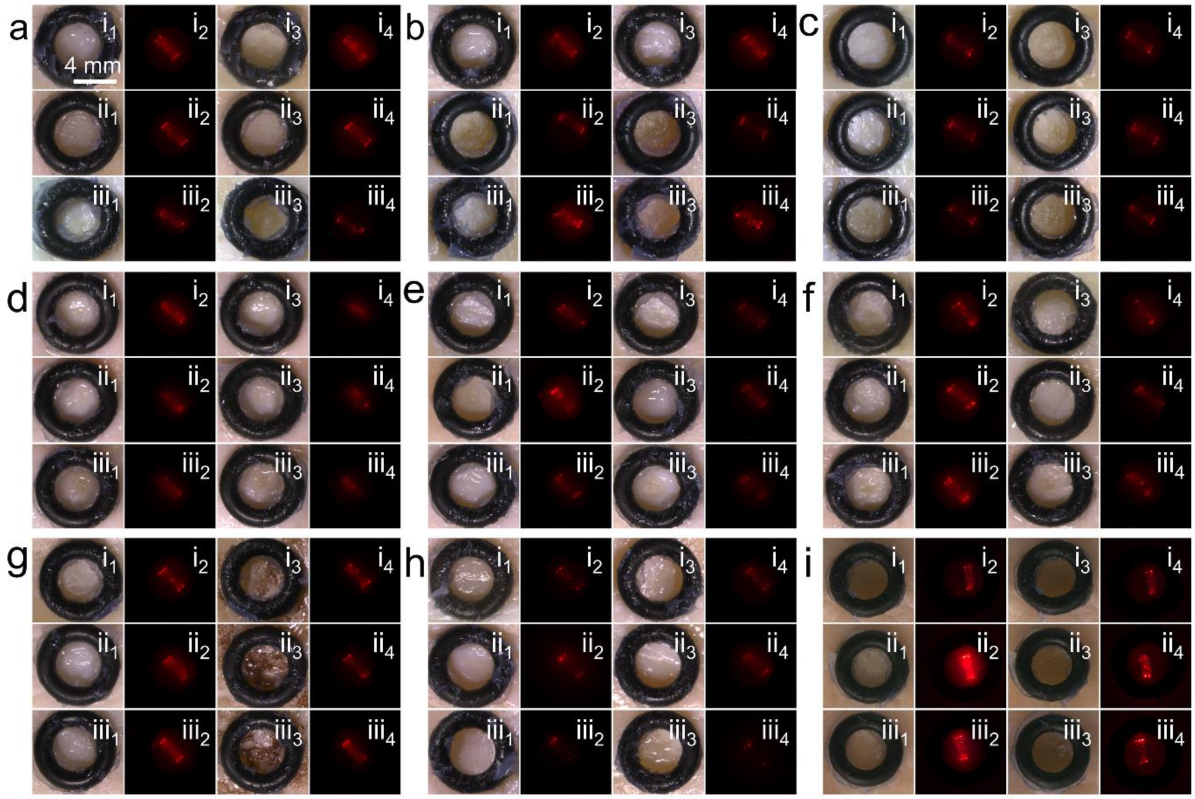

Figure S12. Anti-interference performance of PEI_{600}-UCNPs marking on the porcine skin. (a) Dye-yellow. (b) Dye-carmine. (c) Dye-pink. (d) Fluorescent ink. (e) Toner. (f) Cream. (g) Hair dye. (h) Sunscreen. (i) Perfume. 1–2: Bright and dark fields after dropping PEI_{600}-UCNPs; 3–4: Bright and dark fields with the interference. i–iii: Three repetitions.

Table S1. The energy of OA and PEI ligands on $NaYF_4$ (111) plane.

	(111)/OA	(111)/PEI	(111)	OA	PEI
Energy (eV)	-80637.01	-79823.61	-78467.44	-2169.86	-1355.23

Table S2. Cytotoxicity of PEI_{600}-UCNPs in HaCaT cells at different concentrations.

No.	Concentrations (μg mL^{-1})	Inhibition (%)
1	50 μg mL^{-1}	< 1
2	100 μg mL^{-1}	< 1
3	250 μg mL^{-1}	20.93 ± 0.96

References:

[1] N. Bogdan, F. Vetrone, G. A. Ozin, J. A. Capobianco, *Nano Lett.* **2011**, *11*, 835.

[2] B. Yu, K. Ai, L. Lu, *Appl. Mater. Today* **2020**, *18*, 100493.

[3] R. OFernandes, N. R. Smyth, O. L. Muskens, S. Nitti, A. Heuer-Jungemann, M. R. Ardern-Jones, A. G. Kanaras, *Small* **2015**, *11*, 713.

[4] S. J. Clark, M. D. Segall, C. J. Pickard, P. J. Hasnip, M. I. J. Probert, K. Refson, M. C. Payne, *Z. Kristallogr.* **2005**, *220*, 567.

[5] a) M. J. Abraham, T. Murtola, R. Schulz, S. Páll, J. C. Smith, B. Hess, E. Lindahl, *SoftwareX* **2015**, *1-2*, 19; b) S. Pronk, S. Páll, R. Schulz, P. Larsson, P. Bjelkmar, R. Apostolov, M. R. Shirts, J. C. Smith, P. M. Kasson, D. van der Spoel, B. Hess, E. Lindahl, *Bioinformatics* **2013**, *29*, 845.

[6] N. Schmid, A. P. Eichenberger, A. Choutko, S. Riniker, M. Winger, A. E. Mark, W. F. van Gunsteren, *Eur. Biophys. J.* **2011**, *40*, 843.

[7] A. K. Malde, L. Zuo, M. Breeze, M. Stroet, D. Poger, P. C. Nair, C. Oostenbrink, A. E. Mark, *J. Chem. Theory Comput.* **2011**, *7*, 4026.

[8] C. I. Bayly, P. Cieplak, W. Cornell, P. A. Kollman, *J. Phys. Chem.* **1993**, *97*, 10269.

[9] P. J. Stephens, F. J. Devlin, C. F. Chabalowski, M. J. Frisch, *J. Phys. Chem.* **1994**, *98*, 11623.

[10] a) Harihara.Pc, J. A. Pople, *Theor. Chem. Acc.* **1973**, *28*, 213; b) W. J. Hehre, R. Ditchfield, J. A. Pople, *J. Chem. Phys.* **1972**, *56*, 2257.

[11] M. J. Frisch, G. W. Trucks, H. B. Schlegel, G. E. Scuseria, M. A. Robb, J. R. Cheeseman, G. Scalmani, V. Barone, G. A. Petersson, H. Nakatsuji, X. Li, M. Caricato, A. V. Marenich, J. Bloino, B. G. Janesko, R. Gomperts, B. Mennucci, H. P. Hratchian, J. V. Ortiz, A. F. Izmaylov, J. L. Sonnenberg, Williams, F. Ding, F. Lipparini, F. Egidi, J. Goings, B. Peng, A. Petrone, T. Henderson, D. Ranasinghe, V. G. Zakrzewski, J. Gao, N. Rega, G. Zheng, W. Liang, M. Hada, M. Ehara, K. Toyota, R. Fukuda, J. Hasegawa, M. Ishida, T. Nakajima, Y. Honda, O. Kitao, H. Nakai, T. Vreven, K. Throssell, J. A. Montgomery Jr., J. E. Peralta, F. Ogliaro, M. J. Bearpark, J. J. Heyd, E. N. Brothers, K. N. Kudin, V. N. Staroverov, T. A. Keith, R. Kobayashi, J. Normand, K. Raghavachari, A. P. Rendell, J. C. Burant, S. S. Iyengar, J. Tomasi, M. Cossi, J. M. Millam, M. Klene, C. Adamo, R. Cammi, J. W. Ochterski, R. L. Martin, K. Morokuma, O. Farkas, J. B. Foresman, D. J. Fox, Wallingford, CT 2016.

[12] T. Lu, F. Chen, *J. Comput. Chem.* **2012**, *33*, 580.

[13] W. Humphrey, A. Dalke, K. Schulten, *J. Mol. Graphics Modell.* **1996**, *14*, 33.

[14] G. Bussi, D. Donadio, M. Parrinello, *J. Chem. Phys.* **2007**, *126*, 014101.

[15] H. J. C. Berendsen, J. P. M. Postma, W. F. v. Gunsteren, A. DiNola, J. R. Haak, *J. Chem. Phys.* **1984**, *81*, 3684.

[16] T. Darden, D. York, L. Pedersen, *J. Chem. Phys.* **1993**, *98*, 10089.

[17] M. Parrinello, A. Rahman, *J. Appl. Phys.* **1981**, *52*, 7182.
